# Upconversion Nanocomposite Materials With Designed Thermal Response for Optoelectronic Devices

**DOI:** 10.3389/fchem.2019.00083

**Published:** 2019-03-04

**Authors:** Eduardo D. Martínez, Carlos D. S. Brites, Luís D. Carlos, Ricardo R. Urbano, Carlos Rettori

**Affiliations:** ^1^“Gleb Wataghin” Institute of Physics (IFGW), University of Campinas (UNICAMP), Campinas, Brazil; ^2^Physics Department and CICECO-Aveiro Institute of Materials, University of Aveiro, Aveiro, Portugal; ^3^CCNH, Universidade Federal do ABC (UFABC), Santo André, Brazil

**Keywords:** upconversion nanoparticles, polymer nanocomposites, luminescent coatings, optical thermometry, electrochromic devices, thermochromism, optoelectronic devices, thermoplasmonics

## Abstract

Upconversion is a non-linear optical phenomenon by which low energy photons stimulate the emission of higher energy ones. Applications of upconversion materials are wide and cover diverse areas such as bio-imaging, solar cells, optical thermometry, displays, and anti-counterfeiting technologies, among others. When these materials are synthesized in the form of nanoparticles, the effect of temperature on the optical emissions depends critically on their size, creating new opportunities for innovation. However, it remains a challenge to achieve upconversion materials that can be easily processed for their direct application or for the manufacture of optoelectronic devices. In this work, we developed nanocomposite materials based on upconversion nanoparticles (UCNPs) dispersed in a polymer matrix of either polylactic acid or poly(methyl methacrylate). These materials can be processed from solution to form thin film multilayers, which can be patterned by applying soft-lithography techniques to produce the desired features in the micro-scale, and luminescent tracks when used as nanocomposite inks. The high homogeneity of the films, the uniform distribution of the UCNPs and the easygoing deposition process are the distinctive features of such an approach. Furthermore, the size-dependent thermal properties of UCNPs can be exploited by a proper formulation of the nanocomposites in order to develop materials with high thermal sensitivity and a thermochromic response. Here, we thus present different strategies for designing optical devices through patterning techniques, ink dispensing and multilayer stacking. By applying upconverting nanocomposites with unique thermal responses, local heating effects in designed nanostructures were observed.

## Introduction

Upconversion materials are the subject of intense study both from the fundamental physics point of view and because of their potential applications in various fields of technology (Nadort et al., 2016; Zhou et al., 2018a). They can be synthesized in the form of nanoparticles with controlled composition, morphology, size, crystalline structure and surface chemistry (Wang and Liu, 2008; Bettinelli et al., 2015). The size-dependent thermal properties of upconversion nanoparticles (UCNPs) have recently received much attention. It was shown that below a critical size of about 30 nm, the emission intensity is enhanced as the temperature increases, while thermal quenching occurs for bigger particles (Li et al., 2014). This opens up new opportunities for applications where the size-dependent thermal properties of UCNPs can act as an extra degree of freedom for controlling the optical emissions (Shao et al., 2017). However, in the attempt to apply these materials in the manufacture of optoelectronic devices, the main challenge lies in the method by which UCNPs are to be deposited and integrated into fabrication processes, ensuring a uniform distribution with controlled spatial location. Methods need to be simple, yet sufficiently versatile to be adapted to serial production or to achieve specific architectures. A common strategy to achieve this goal is the formulation of nanocomposite materials.

The topic of hybrid materials formed by inorganic nanoparticles in polymer matrices has been addressed in several review papers (Binnemans, 2009; Kango et al., 2013; Bai et al., 2016; Pastoriza-Santos et al., 2018). Luminescent NPs have already been included in polymers by polymerization of different monomers, which may or may not be attached to the NPs by covalent bonds. A common strategy for this is the polymerization of methacrylate monomers catalyzed by 2,2′-azo-bisisobutyronitrile (AIBN). This approach has successfully been applied to include QDs (Lee et al., 2000; Sun et al., 2008; Guan et al., 2009), plasmonic nanoparticles (Pastoriza-Santos et al., 2018) and upconverting crystals (Wang et al., 2007; Tabanli et al., 2017; Zhang et al., 2018). Although this strategy is well-suited for obtaining monoliths and molded pieces with excellent optical properties, it is not appropriate for direct application of the nanocomposites in forming coatings or microstructures. On the other hand, luminescent inks composed by inorganic nanoparticles dispersed in solutions with different additives (including polymers) were developed in the past by the groups of May (Blumenthal et al., 2012; Meruga et al., 2012, 2014), Xu (You et al., 2015) and others (Tan et al., 2010; Furasova et al., 2017; Ma et al., 2017).

Here, we advance the development of simple, yet reliable polymer-based nanocomposite materials as a convenient and versatile way to deposit temperature sensitive UCNPs on rigid and flexible substrates. This allows the formation of homogeneous coatings, multilayer structures and the application of soft-lithography techniques, to form luminescent patterns. We explored two different polymers to act as a matrix material: polylactic acid (PLA) and poly(methyl methacrylate) (PMMA). The former constitutes a biodegradable polymer, which is increasingly used as a feeding material for additive manufacturing (3D printing) becoming affordable and accessible; the latter, on the other hand, presents a higher thermal and mechanical stability. In addition, PMMA can be used as a positive tone resist for electron beam lithography (EBL) for fabrication of patterns in the nanoscale. Both polymers are soluble in chloroform, a fast evaporating solvent that can also act as a dispersion medium for UCNPs; therefore, the integration of both materials is straightforward. As a distinctive feature, we take advantage of the size-dependent thermal properties of UCNPs by combining particles with different sizes and compositions. The thermo-sensitive nanocomposites were tested as probes for heat dissipation in two kinds of nanostructures: first, dry deposits of gold nanostars (AuNSs) where the thermoplasmonic effect takes place. Second, by assembling an electrothermal device using percolating networks of silver nanowires (AgNWs) (Martínez et al., 2016, 2018a, 2019). In the former nanostructure, when the local surface plasmon resonance (LSPR) of AuNSs is excited, energy is dissipated via electron-phonon scattering generating heat (Baffou et al., 2010; Rodríguez-Oliveros and Sánchez-Gil, 2012). Therefore, a local increase in temperature can be triggered externally by light. In fact, the spectral position of the LSPR in AuNSs can be tuned during the synthesis to match the wavelength of the light used to excite the upconversion luminescence in UCNPs. In the case of AgNWs networks, this is a system of increasing technological interest. Among other applications, they are used in transparent electrodes (Anh Dinh et al., 2013; Ye et al., 2014), solar-cells (Langley et al., 2014), flexible and wearable electronics (Myers et al., 2015; Lee et al., 2017). Moreover, upon electrical currents flows, heat is dissipated due to the Joule effect. Therefore, AgNWs networks are also used as nanoheater films (Kim et al., 2013; Huang et al., 2015) being an ideal system for testing the thermometric nanocomposites developed here.

In this work, we show some of the potential uses of the polymer-UCNPs composite materials by performing neat examples of microfabrication, multichromatic inks and multilayers, and thermally sensitive optical coatings. We are certain that this can provide new routes for the integration of nanoparticles in the development of optoelectronic devices and luminescence-based technologies.

## Experimental Section

### Materials

Sodium trifluoroacetate 98% (CAS 2923-18-4), yttrium(III) trifluoroacetate >99.99% (CAS 304851-95-4), erbium(III) oxide (CAS 12061-16-4), ytterbium(III) oxide (CAS 1314-37-0), thulium(III) oxide (CAS 12036-44-1), trifluoroacetic acid 99% (CAS 76-05-1), gold(III) chloride trihydrate >99.9% (CAS 16961-25-4), AgNO_3_ 99.9999% (CAS 7761-88-8), L-ascorbic acid, reagent grade (CAS 50-81-7), trisodium citrate dihydrate (CAS 6132-04-3), mercaptosuccinic acid 97% (CAS 70-49-5), poly(sodium 4-styrenesulfonate) MW ~70 000 g mol^−1^ (PSS, CAS 25704-18-1), ethylene glycol (EtGOH) anhydrous (99.8%), polyvinylpyrrolidone (PVP) 360,000 mol. wt., iron(III) chloride 97% (CAS 7705-08-0), oleic acid (OA, technical grade, 90%) and 1-octadecene (ODE, technical grade, 90%) were purchased from Sigma-Aldrich Co. Gadolinium(III) acetate hydrate 99.9% (CAS 100587-93-7) and Cerium(III) chloride heptahydrate 99% (CAS 18618-55-8) were purchased from Alfa Aesar. PMMA 7% wt. in chlorobenzene resist (PMMA C7) was purchased from MicroChem Corp, USA. All commercial reagents were used without further purification. Rare-earth acetates and trifluoroacetates not listed above were prepared in our laboratory using the corresponding rare-earth oxides (X_2_O_3_, X = Yb, Tm, Er). In a 500 mL round bottom flask containing 30–50 mL of a 50% (v/v) aqueous solution of either acetic acid or trifluoroacetic acid, 1–2 g of the respective oxide was added. The mixture was refluxed for 1–2 h until the complete dissolution of the oxides resulted in a clear solution. The solution was then transferred to a Pyrex open vessel and maintained at 60°C to evaporate the liquid. The dried precipitates of acetate/trifluoroacetate salts were extracted.

### Synthesis of UCNPs

Large (300 nm) pure hexagonal phase NaYF_4_ nanoparticles co-doped with ytterbium (20 mol%) and erbium (2 mol%) were synthesized by the well-established thermal decomposition method of fluoroacetates, adapting protocols described by Ye and co-workers (Ye et al., 2010). In a three neck 200 mL round bottom flask containing 15 mL of ODE and 15 mL of OA, a total of 6.2 mmol of NaCOOCF_3_, 2.6 mmol of Y(COOCF_3_)_3_, 0.68 mmol of Yb(COOCF_3_)_3_ and 0.068 mmol of Er(COOCF_3_)_3_ were added. The flask was sealed and heated under vacuum at 125°C in order to dissolve the precursors and degas the solution. After 1 h, a condenser was mounted, and the temperature was rapidly increased (10–20°C·min^−1^) up to 330°C under an argon flux. After 30–35 min from the beginning of the heating stage, the reaction flask was retired from the mantle and 15 mL of ODE were added to quench the reaction. Extraction of the UCNPs was performed by adding an ethanol: hexane 4:1 v/v mixture and centrifugation for 5 min at 2,400 rpm, corresponding to a relative centrifugal force (RCF) of 1,004 RCF. The washing procedure was repeated four times and the UCNPs were finally dispersed in hexane with a final concentration of about 2 g·cm^−3^. Same method was applied for the synthesis of additional large-sized UCNPs with compositions detailed in Table 1.

**Table 1:** Nomenclature, nominal composition and particle size ± std (determined using the TEM images) of the UCNPs employed in the formulation of the nanocomposites.

**Sample**	**Composition**	**Size (nm)**	**Obs**.
1S	NaGd_0.695_Yb_0.300_Tm_0.005_F_4_@NaGdF_4_	8.8 ± 0.8	Small
2S	NaGd_0.78_Yb_0.20_Er_0.02_F_4_@NaGdF_4_	11.0 ± 1.4	
3S	NaGd_0.63_Yb_0.20_Ce_0.15_Ho_0.02_F_4_@NaGdF_4_	8.0 ± 1.3	
1L	NaY_0.695_Yb_0.300_Tm_0.005_F_4_	(400 ± 15) × (120 ± 10)	Large
2L	NaY_0.78_Yb_0.20_Er_0.02_F_4_	(300 ± 8) × (160 ± 6)	
3L	NaY_0.63_Yb_0.20_Ce_0.15_Ho_0.02_F_4_	(85 ± 5) × (40 ± 3)	

Small size hexagonal core-UCNPs (<12 nm) were synthesized by the co-precipitation route adapting the protocols reported by Wang and co-workers (Wang et al., 2014) using rare-earth acetates as the main precursors. A total of 1 mmol of X(COOCH_3_)_3_ (X = Gd, Yb, Tm, Er) was added in a 200 mL round bottom flask containing 15 mL of ODE and 9 mL of OA. The nominal molar ratio of each system was 1:0.78:0.2:0.02 Na:Gd:Yb:Er and 1:0.695:0.3:0.005 Na:Gd:Yb:Tm. The mixture was heated up to 160°C for 1 h to dissolve the precursors and then cooled down to room temperature. At this point, a freshly prepared mixture containing 2.5 mL of a 1 M NaOH methanol solution and 10.1 mL of 0.4 M NH_4_F solution in methanol was rapidly injected. The flask was heated to 50°C for 30 min and then sealed. The temperature was raised to 100°C and the vacuum pump was connected. After 15 min, the vacuum pump was shut down, a condenser was mounted, and the temperature was increased to 280°C under argon flux. The flask was retired from the mantle after 1 h and allowed to cool down to room temperature. Extraction of the UCNPs was performed by adding excess anhydrous ethanol and centrifugation using 15 mL Falcon tubes. Centrifugation was performed at 1,004 RCF (2,400 rpm) for 7 min. The precipitated UCNPs were redispersed in 4 mL of cyclohexane and ethanol was added to complete the volume. The centrifugation and washing procedure was repeated twice. Finally, the UCNPs were dispersed in 8 mL of cyclohexane. As the quantum yield of the UC process is lower for smaller particles due to surface-related quenching processes, a core–shell structure was formed by growing a thin inert layer on the surface of the pre-synthesized core UCNPs. For this, 1 mmol of Gd(COOCH_3_)_3_ was added to 8 mL of OA and 12 mL of ODE in a 200 ml three-neck round bottom flask. The mixture was heated up to 160°C for 1 h and then cooled down to room temperature. Then, 6 mL of the core UCNPs colloid in cyclohexane were added. The subsequent addition of a methanol solution of NaOH and NH_4_F and the following procedures were identical to those previously described for the synthesis of core nanoparticles. The same protocol was used for the synthesis of small-sized UCNPs doped with Ce^3+^/Ho^3+^ as detailed in Table 1.

### Synthesis of AuNSs

Gold nanostars (AuNSs) were synthesized following a seed mediated protocol at room temperature. Gold nanoparticles (AuNPs) were first synthesized to be used as seeds by adding 15 mL of a 1 wt.% trisodium citrate aqueous solution into 100 mL of a boiling solution of gold(III) chloride 1 mM and maintained at boiling temperature for 15 min. When cooled down to room temperature, the resulting colloid was filtered through a 0.22 μm PES syringe filter. For the synthesis of AuNSs, 20 μL of a 1 M HCl solution were added to 20 mL of 0.25 mM gold(III) chloride solution in Milli-Q ultrapure water. 200 μL of the seed AuNPs were added and stirred at 800 rpm for 3 min in a 100 mL round bottom flask. Immediately afterwards, 200 μL of 3.3 mM AgNO_3_ and 100 μL of 0.1 M L-ascorbic acid solutions were added quickly and simultaneously to the reactor vessel. After 1 min, 50 μL of a 1 mM mercaptosuccinic acid solution was added followed by 1 mL of a 1 wt.% PSS solution. All steps described before were performed at ambient temperature. It is worth mentioning that by varying the ratio between the ascorbic acid, the AgNO_3_ added and the acid concentration in the reactor, it is possible to tune the branching growth of the resulting AuNSs and therefore the spectral position of the surface plasmon resonance. Here, concentration values were tuned to produce AuNSs with a plasmon resonance in the 900–1,000 window, matching the wavelength of the excitation light used for upconversion experiments. Further details of the procedure and the influence of the synthesis parameters in the resulting AuNSs can be found in Yuan and co-workers (Yuan et al., 2012).

### Synthesis of AgNWs

Silver nanowires (AgNWs) were synthesized by adapting the method described by Jiu and co-workers (Jiu et al., 2014). Briefly, 0.2 g of PVP (360,000 g·mol^−1^) were dissolved in 15 mL of anhydrous EtGOH and a solution containing 0.25 g of AgNO_3_ in 10 mL of EtGOH was added at room temperature simultaneously with 3.25 mg of a 0.6 mM FeCl_3_ solution in EtGOH. The final mixture was poured into a 100 mL round flask and sealed. The flask was then placed into an oven at 130°C for 5 h without agitation. After cooling down to room temperature, extraction of the AgNWs was performed by adding excess volume of acetone. The mixture was centrifuged at 1,000 rpm and the precipitates were dispersed in isopropanol. This procedure was repeated three times until a final dispersion of AgNWs in isopropanol was prepared in a 2.5 g·L^−1^ mass concentration.

### Materials Characterization

UCNPs, AuNSs and AgNWs were characterized using optical and electron microscopy. Electron microscopy was performed at LNNano, CNPEM, Campinas, Brazil. SEM images were acquired in a FEI Quanta 650 FEG microscope operated at 20 kV. Transmission electron microscopy (TEM) images were obtained with a JEM 2,100 (JEOL) equipped with a LaB_6_ filament and operated at 200 kV. X-ray diffraction (XRD) was performed in a Phaser D2 diffractometer (Bruker) while dynamical light scattering (DLS) was performed in a Nanopartica SZ-100 DLS (Horiba). UV-Visible spectrophotometry was performed in a Cary 8454 Agilent Technologies. Profilometry measurements were performed at LAMULT-IFGW using a Dektak 150, Veeco stylus profilometer. Upconversion was recorded using as excitation a collimated BL976-PAG900 FBG stabilized laser (ThorLabs). All measurements were performed at a nominal power of 500 mW, except otherwise indicated. The emission was analyzed with a QEPro spectrometer (Ocean Optics) coupled to a 600 μm diameter optical fiber. A short-pass optical filter was used to avoid the spectrometer saturation by the 976 nm laser. The thermal response of UCNPs was studied by depositing the upconverting material on silicon substrates. This was carried out in two ways: the formulated nanocomposites were spin-coated at 3,000 rpm, while dry deposits of UCNPs were formed by drop-casting the colloids and evaporating the solvent (hexane or cyclohexane) at 130°C. The samples were placed in thermal contact on a Peltier plate controlled by an Arduino board. The emission spectra were acquired at varying temperatures of the plate. Spin-coating was also used to deposit nanocomposite films on glass substrates for transmittance and profilometry characterization.

## Results and Discussion

**Figure 1:**
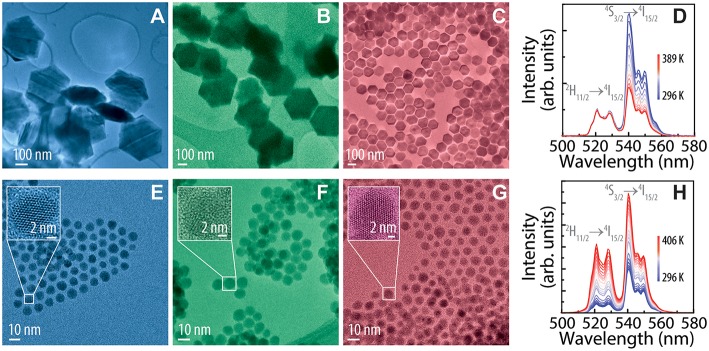
TEM images of **(A–C)** large-size and **(E–G)** small-size UCNPs doped with **(A,E)** Tm^3+^, **(B,F)** Er^3+^ and **(C,G)** Ce^3+^-Ho^3+^. Temperature dependent emission in the green spectral range of **(D)** 2L and **(H)** 2S UCNPs under 976 nm excitation.

Two sets of UCNPs were synthesized consisting of small-sized (<20 nm) and large-sized (>50 nm) UCNPs and containing one of the following emitting ions (activators): Tm^3+^, Er^3+^ or Ho^3+^, with main emission lines in the blue, green or red part of the spectrum, respectively. All particles were co-doped with a certain amount of Yb^3+^ ions acting as sensitizer. The composition of each system and the nomenclature adopted here is detailed in Table 1. TEM images of each UCNPs system are shown in Figure 1 and the particle size distributions resulting from the analysis of the images are presented in Supplementary Figure 2. In Supplementary Figure 3, TEM images and partcticle size analysis are shown for UCNPs of the set 2S before and after the formation of the shell layer. X-ray diffraction shows that all particles present a hexagonal (β-) crystal structure (Supplementary Figure 1). UCNPs containing Er^3+^ either large- and small-size (from now on 2L and 2S, respectively) are shown in Figures 1B,F, whereas the emission spectra obtained at calibrated temperatures using a Peltier plate are displayed in Figures 1D,H for each nanoparticle system. The characteristic parameters resulting from the thermal calibration are summarized in Supplementary Table 1. The thermal dependence of the emission spectra for additional UCNPs studied in this work is presented in the Supplementary Figure 5. In all cases, the emission intensity is thermally quenched for large-sized UCNPs and thermally enhanced for small-sized UCNPs. This is in agreement with the size-dependent thermal response of UCNPs as already reported by Li and collaborators (Li et al., 2014). On one side, the thermal quenching effect for large-sized UCNPs is well-known and typically associated with non-radiative multiphonon relaxation (Shen et al., 2010; Yu et al., 2016). However, the thermal enhancement effect that takes place in small-size UCNPs was reported only recently and a conclusive explanation is still lacking. So far, quantum size effects affecting the phonon density of states (Li et al., 2014), thermal desorption of water molecules (Shao et al., 2017), and energy transfer between Yb^3+^ ions and anchoring groups of the capping molecules (Zhou et al., 2018b), have been proposed as an explanation for this anomalous effect. Although at this time we can not add new information to solve this issue, we can take advantage of the thermal responses by formulating nanocomposites combining both kinds of UCNPs.

### UCNPs-Polymer Nanocomposites

**Figure 2:**
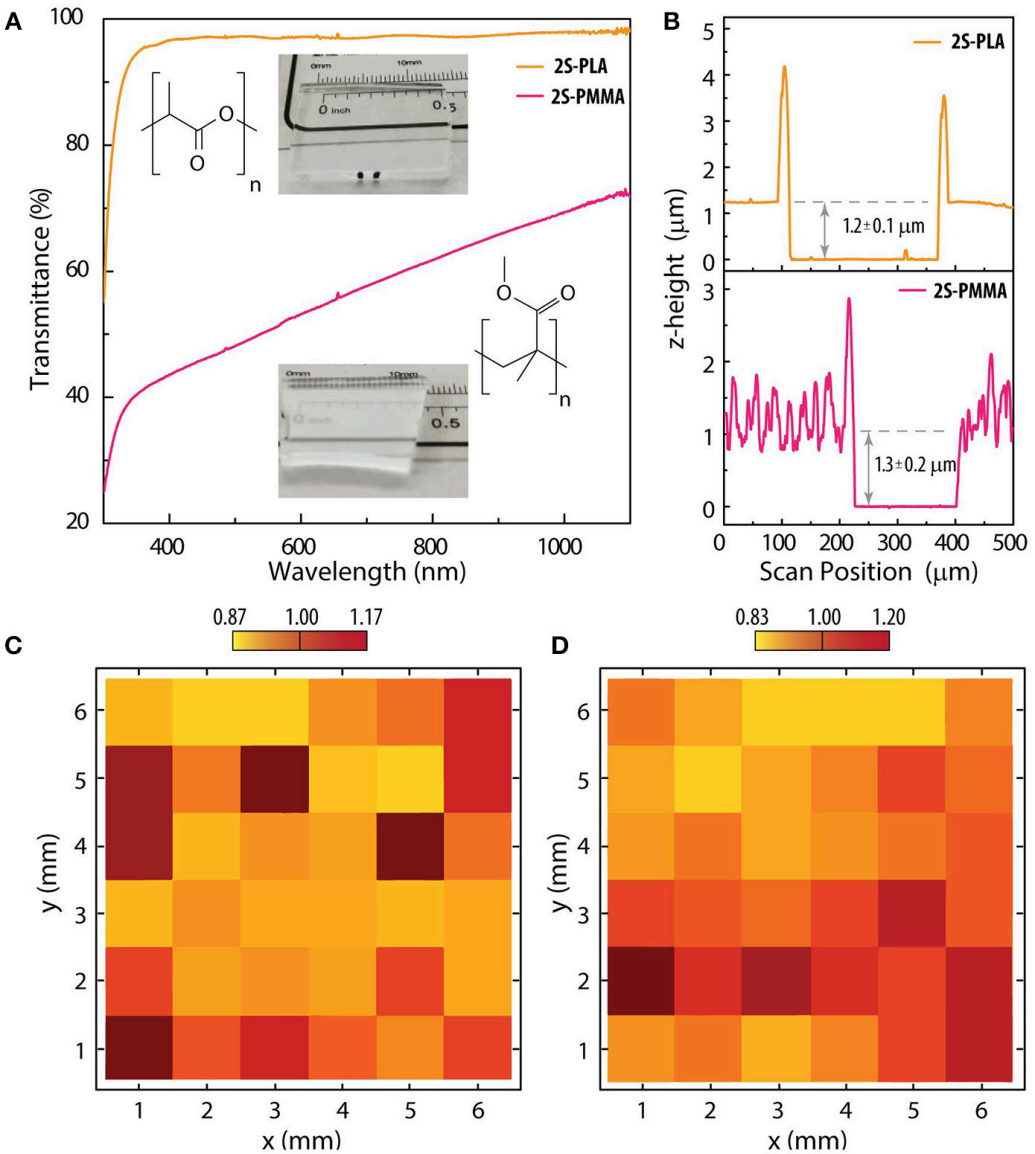
Nanocomposites containing small-size UCNPs doped with Yb^3+^-Er^3+^ deposited on glass substrates by spin-coating at 3,000 rpm. **(A)** Optical transmittance and pictures of 2S-PMMA and 2S-PLA samples. **(B)** Stylus profilometry performed on a scratch made on the films. Homogeneity of integrated upconversion emission spectra for nanocomposites based on **(C)** 2S-PLA and **(D)** 2S-PMMA.

For preparation of the nanocomposites, a certain amount of the colloids formed by UCNPs in cyclohexane was placed in a glass vial under a gentle flux of N_2_ until complete evaporation of the solvent. The dried UCNPs were dispersed either in pure chloroform or in a 2 %wt. PLA solution in chloroform by means of vortex agitation and ultrasonic bath. For PMMA nanocomposites, equal amounts of the PMMA resist and the chloroform-UCNPs colloid were mixed. The approximate concentration of particles in the polymer solutions were ~0.04 g·mL^−1^ for small-size UCNPs and ~0.0015 g·mL^−1^ for large-size UCNPs. The prepared nanocomposite solutions were spin-coated at 3,000 rpm on glass substrates, forming a thin uniform layer. In order to compare the PMMA and PLA based films, the optical transmittance, the integrated intensity of the upconversion emissions and the thickness of the films were measured in samples containing 2S UCNPs. The optical transmittance was measured using a UV-visible spectrophotometer in the transmission mode (Figure 2A) showing a much higher transparency for the case of PLA films, while PMMA films present an opaque aspect. This could be due to the content of chlorobenzene in the PMMA resist used, which has a slower evaporation rate (1.07 compared to butyl acetate) than that of chloroform (11.6 compared to butyl acetate). The evaporation of chloroform could induce a coarse-grained deposition of the polymer. It is worth mentioning that the surface topography is not due to a clustering of the UCNPs as similar optical haze is observed for films without UCNPs. Also, it was observed that reducing the fraction of chloroform in the UCNPs-PMMA solution improves the transparency, although the viscosity is increased and therefore, the film thickness after coating is higher. The thickness of the films were characterized by stylus profilometry on a scratch made *ad-hoc* (Figure 2B), showing similar values for each film (~1.2 μm for PLA and ~1.3 μm for PMMA). The roughness was also analyzed finding values of root mean square roughness (*R*_q_) of 0.005 μm and 0.37 μm for PLA and PMMA films, respectively. The higher *R*_q_ observed for the case of PMMA films is consistent with the lower transmittance discussed previously. The material removed from the scratches accumulated on the edges causing the peaks observed in the profilometry scans. In order to analyze the homogeneity of the coverage with UCNPs, the samples were mounted on a *x-y* micro-positioner stage. The emission spectra under excitation with a 976 nm collimated laser beam (ca. 1 mm spot size) was registered at different positions scanning a square region of 6 × 6 mm^2^. A 2D map was constructed (Figures 2C,D) by integrating the emission spectrum at each point. Normalizing by the mean value, the emission intensity remains under a 15–20% deviation for the PLA and PMMA films, respectively.

**Figure 3:**
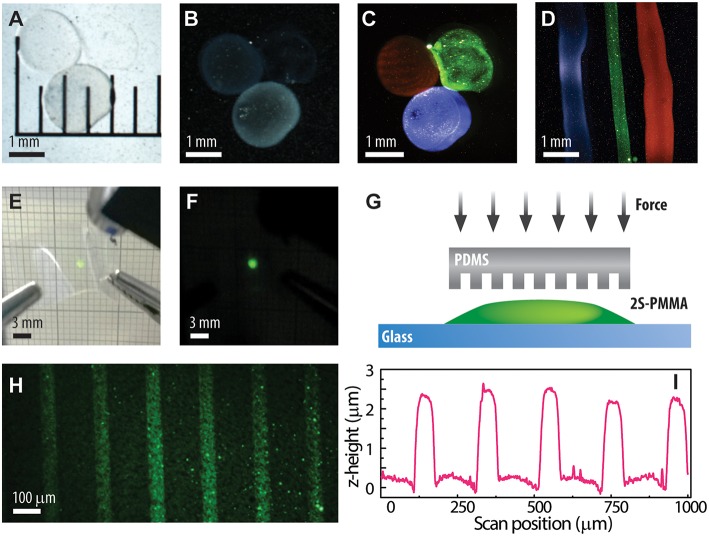
Strategies to apply the PLA based nanocomposites as ink to directly write multicolor luminescent features. Optical images under **(A)** bright field, **(B)** dark field and **(C,D)** NIR light illumination. **(E,F)** 2L-PLA nanocomposite deposited on a flexible substrate. Scheme **(G)** of the soft lithography process to transfer micro-patterns. Luminescent tracks produced by soft lithography on 2L-UCNPs in PMMA under **(H)** NIR illumination and **(I)** height profile revealed by stylus profilometry.

The development of polymer-based nanocomposites is driven by the necessity of controlling the integration of nanoparticles into functional surfaces and structures. With that purpose, we tested the direct application of the formulated nanocomposites in different ways. On one side, the PLA based nanocomposite solutions containing large-size UCNPs emitting in the blue (1L, Tm^3+^ doped), green (2L, Er^3+^ doped) or red (3L, Ce^3+^/Ho^3+^ co-doped) were used as inks to directly deposit drops and tracks with characteristic luminescent properties. Optical images of the handmade deposits are shown in Figure 3 under white light (bright field, Figure 3A), dark field (Figure 3B) and near infrared (NIR, 976 nm) excitation (Figures 3C,D). Similar results (not shown) were obtained by using the UCNPs-PMMA nanocomposites. Figure 3D shows the use of a PLA-based nanocomposite for drawing luminescent tracks. As PLA is one of the mainstream polymer used in conventional 3D printing, this particular nanocomposite constitutes a step forward. Although we show here a proof of concept by applying the nanocomposite ink manually with a syringe, the deposition may be further improved by adopting controlled injection methods well-established in the additive manufacturing technology (Ligon et al., 2017). Aditionally, the rheology of the composite inks can be optimized for a better control of the dispensing mechanism by varying the mass fraction of the polymers, while the surface tension can be adjusted by adding proper surfactants (Kumar et al., 2016).

The excellent properties of the PLA based nanocomposite can be clearly exposed by spin-coating the 2L-PLA solution into flexible substrates, in this case, a cellulose acetate sheet. The resulting film shows a high transparency and luminescence under NIR excitation as shown in Figures 3E,F.

By using the UCNPs-PMMA nanocomposite, we applied soft lithography methods to produce line patterns in the microscale. For that, a polydimethylsiloxane (PDMS) stamp was first prepared from a Sylgard 184 kit (Dow Chemicals®) following standard protocols (Qin et al., 2010). On a glass slide, 5 μL of the 2L-PMMA nanocomposite was poured; immediately afterwards the PDMS stamp was pressed and held in that position for 5 min, and then placed in a hot plate at 180°C for another 5 min. Once the sample was cooled down to room temperature, the stamp was carefully retired. A scheme of the method is depicted in Figure 3G. The resulting film presents a structure composed of the inverse pattern of the stamp, in this case, with parallel lines, ~60 μm wide, showing a clear contrast in the luminescence under NIR excitation (Figure 3H). The luminescence contrast arises from the topography of the sample as revealed by the profilometry scan shown in Figure 3I. Each line track has a step height of 2.0–2.5 μm. It is worth mentioning that a background luminescence is detected as residual material containing UCNPs remains in the regions of contact between the PDMS and the base glass substrate.

### Thermal Properties of UCNPs-Polymer Nanocomposites

**Figure 4:**
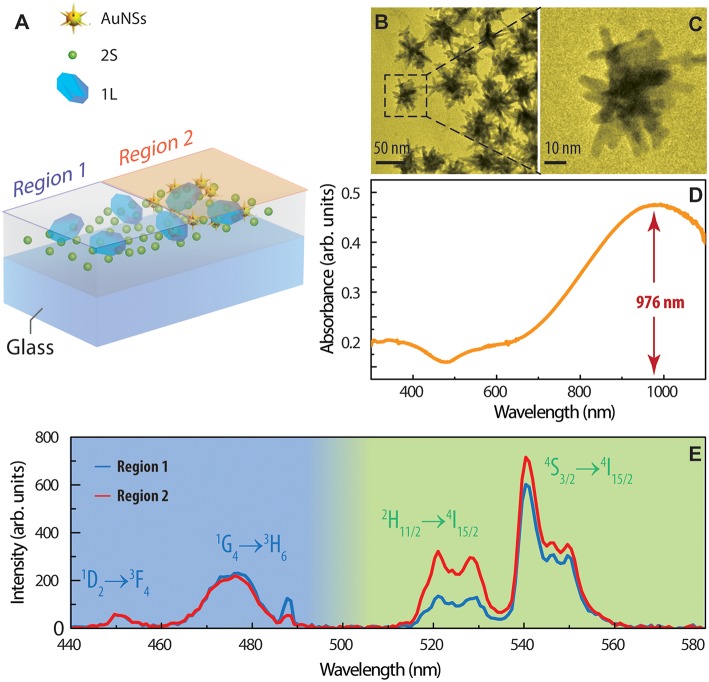
Thermal effects on the emissions of polymer-UCNPs nanocomposites. **(A)** Scheme of samples for the study of the thermoplasmonic effect in AuNSs. **(B,C)** TEM images of AuNSs and **(D)** absorption spectra of AuNSs colloids. **(E)** Emission spectra obtained on regions without (region 1) and with (region 2) AuNSs.

The distinctive thermal properties of small- and large-sized UCNPs can be exploited to produce polymer nanocomposites with unique thermal response. We explored two approaches for the application of the polymer based UCNPs nanocomposites as thermally sensitive materials. For the first approach, depicted in Figure 4A, part of a glass substrate was first covered with AuNSs (Figures 4B,C) by drop-casting. Then, a PLA based nanocomposite containing UCNPs of the set 1L and 2S was spin-coated on top of it; therefore, two regions of the sample can be distinguished and compared: region 1 containing only the UCNPs and region 2 where UCNPs are deposited above AuNSs. The plasmon resonance of AuNSs (Figure 4D), which can be tuned by synthesis, is spectrally located at the NIR region, matching the wavelength of the light used to excite the UCNPs. The absorbed electromagnetic energy dissipates in the form of heat, which is transferred to the UCNPs located above. In the region containing AuNSs (region 2) the intensity of emissions of particles 2S (Er^3+^ doped) is enhanced compared to that measured in region 1 (Figure 4E). However, the emissions of UCNPs 1L is slightly reduced; therefore, the overall color of the total emission is different in both regions due to the presence of AuNSs. For UCNPs containing Er^3+^, the local temperature can be directly calculated from the emission spectra, following the procedures described in the Supplementary Material. By using Equation S1 (Supplementary Material), the temperature in region 2 was determined to be *T* = (92 ± 4)°C while in region 1, *T* = (25 ± 3)°C. It is worth noticing that by using the PLA-based nanocomposites, the homogeneous covering with UCNPs after spin-coating allows a precise comparison between both regions, being an improvement of previous methodologies in which the UCNPs were deposited by drop-casting the colloids and evaporating the solvent (Martínez et al., 2018b). This demonstrates the clear benefits of the nanocomposites formulated in this work.

**Figure 5:**
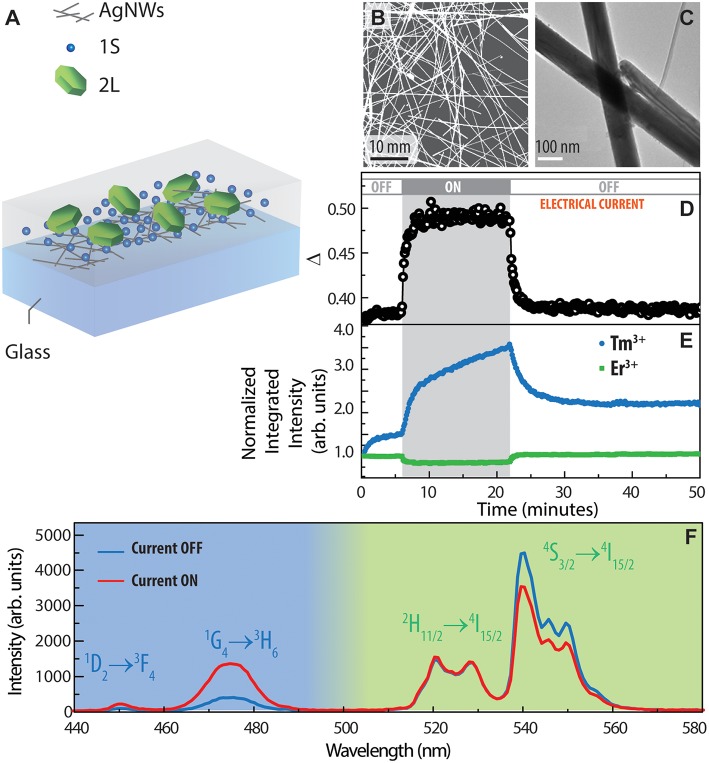
**(A)** Scheme of the electrothermal devices formed by AgNWs and UCNPs nanocomposites. **(B)** SEM and **(C)** TEM images of AgNWs. **(D)** Evolution of the thermometric parameter and **(E)** integrated emissions of blue and green part of the spectrum during an electrothermal action off-on-off with an applied electrical power of 1.3 W. **(F)** Emission spectra of the electrothermal device under states on and off.

In the second approach, we applied the PMMA-based nanocomposite to characterize the heat dissipation in AgNW conductive networks. A transparent thin film formed by a percolating network of AgNWs in PMMA was used as nanoheater film (Figures 5A–C) following previous protocols (Martínez et al., 2016, 2018a). On top of it, a PMMA based nanocomposite containing UCNPs of the set 2L and 1S was deposited by spin-coating. Again, the use of the nanocomposite presented here represents a clear improvement of our previous work (Martínez et al., 2019) by ensuring a homogeneous covering. The resulting device, depicted in Figure 5A, allows for the external electrical control of the temperature of UCNPs by supplying a DC current throughout the AgNWs network, dissipating heat because of the Joule effect. When the power supply (1.3 W) was turned on, a noticeable change in the emission spectra was observed (Figure 5F). While the main emission line coming from the 2L UCNPs is thermally quenched, the emissions arising from UCNPs 1S are thermally enhanced. The integration of the spectra considering the blue band (440–500 nm) and the green band (500–580 nm) shows clearly that the effect of the current flow through the AgNWs film produces an augmented blue emission and a diminished green emission; furthermore, the spectral changes are reversible (Figure 5E). Interestingly, when considering the emissions from Er^3+^, as discussed previously, it is possible to register the thermometric parameter (Figure 5D) throughout the experiment and use it to calculate the effective temperature at the emitter position. The temperature reached during the electrothermal action was (87 ± 3)°C.

### Multilayer Stacking

**Figure 6:**
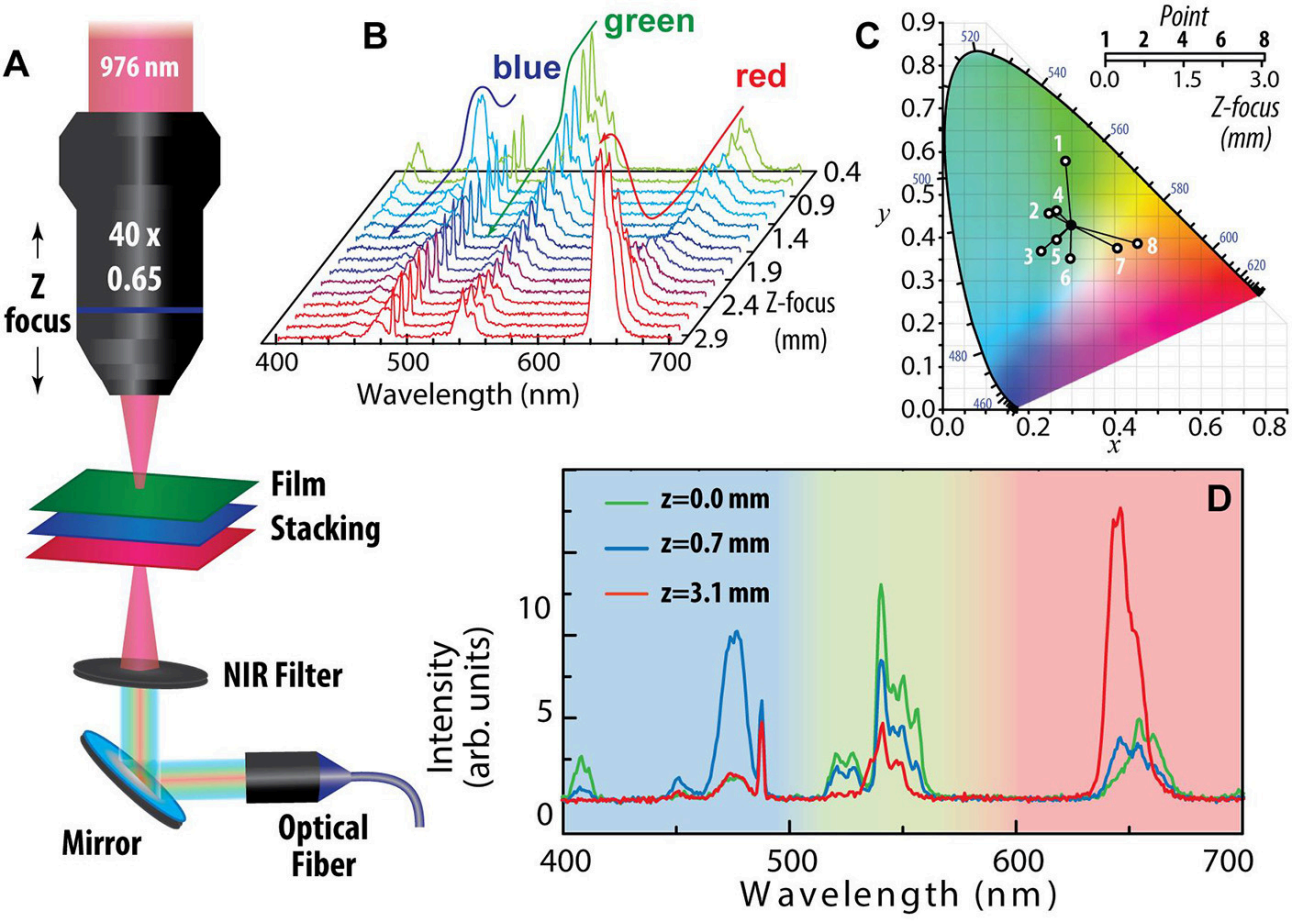
Multicolor structure based on film stacking of UCNPs-PLA layers and z-focusing. **(A)** Scheme of the experimental set-up. **(B)** Emission spectra as a function of the z-focus position. **(C)** CIE 1931 chromaticity diagram showing the emission color obtained at selected z-focus positions. Calculations were made using ColorCalculator v7.15 OSRAM SYLVANIA, Inc. **(D)** Spectra at z-focus positions of maximum green (*z* = 0 mm), blue (*z* = 0.7 mm) and red (*z* = 3.1 mm) emission color.

Finally, to take advantage of the homogeneous coatings formed by spin-coating PLA-UCNPs, we built a multilayer structure formed by stacking PLA films deposited on thin glass coverslips (130–160 μm thick) containing UCNPs emitting in the green (2S), blue (1L) or red (3L) part of the spectrum. When mounting the multilayer stack under an objective lens (40x, NA 0.65), the NIR excitation laser can be focused in a spot of small volume whose position can be adjusted by micrometer screws. By using the set-up shown in Figure 6A we were able to register the overall emission spectra by varying the position of the z-focus (Figure 6B) and observing a clear shift in the emission color from green to blue, and then to red, as the characteristic emission lines of Er^3+^, Tm^3+^, and Ho ^3+^ are successively excited. The change in the emission spectra in the blue (400–500 nm), green (500–600 nm) and red (600–700 nm) bands at each z-focus position (Figure 6D) modified the overall color of the emitted spectra, as shown in a chromaticity diagram Commission Internationale de l'Éclairage (CIE) 1931 on Figure 6C.

Notice that the multilayer stacking concept displayed in this section can be improved and adapted for other systems. For example, by forming stacking of UCNPs-nanocomposites on thin or flexible substrates, membranes can be produced whose deformation could be followed optically.

## Conclusion

Polymer based nanocomposites containing UCNPs with different sizes and compositions were developed. Two polymers were used as matrix materials: PMMA and PLA, both of which are soluble in chloroform, forming stable suspensions upon the addition of UCNPs with oleic acid capping. The nanocomposite solutions can be deposited by spin-coating on several substrate materials, including flexible polymer sheets, producing homogeneous films (~1 μm thick) with uniform upconversion luminescence. The nanocomposite solutions can also be applied directly, resembling an ink, providing a simple way to draw tracks and features with a precision mainly limited by the dispensing method. By using the nanocomposite inks in combination with PDMS stamps and soft lithography techniques it is possible to transfer microscale patterns forming luminescent structures. Furthermore, we take advantage of the size-dependent thermal properties of UCNPs by formulating nanocomposites containing different combinations of small- and large-size UCNPs. We have successfully tested the thermally sensitive materials developed here by applying the nanocomposites of thermally active nanostructures. Two approaches were adopted, first, by using AuNSs with remarkable photothermal conversion efficiencies due to the thermoplasmonic effect; and second, by using conductive networks of AgNWs that can act as nanoheater films when DC currents are supplied. We successfully probed the external control of the intensity of emission lines due to the local change in the temperature, which could be directly measured by following the emissions of Er^3+^ in the green part of the spectrum or directly detected as a change in the emission color. The versatility of the nanocomposites was explored by constructing a multilayer stacking in combination with focusing optics, a simple set-up that showed to be highly sensitive to vertical displacements resulting in clear changes in the emission color. With these advances, we demonstrate that nanocomposites colloids presented in this work are extremely useful materials for direct application of UCNPs, and possibly other nanomaterials, in the assembling of novel photonic and optoelectronic devices.

## Data Availability

The raw data supporting the conclusions of this manuscript will be made available by the authors, without undue reservation, to any qualified researcher.

## Author Contributions

EM conceived the project, synthesized the particles and performed all measurements. CB and LC discussed the project, analyzed the data, performed the calculations of thermometry and prepared all figures. RU and CR contributed to the experimental set-up and discussion of the project. The manuscript was written with contributions from all authors.

### Conflict of Interest Statement

The authors declare that the research was conducted in the absence of any commercial or financial relationships that could be construed as a potential conflict of interest.
