# Supplementary material for: Upconversion Nanocomposite Materials With Designed Thermal Response for Optoelectronic Devices
Source: Front Chem. 2019 Mar 4;7:83. doi: 10.3389/fchem.2019.00083 (PMC6410674; doi:10.3389/fchem.2019.00083)
Supplement: Supplementary file 1 [file Data_Sheet_1.pdf]

Supplementary Material

Upconversion Nanocomposite Materials with Designed Thermal Response for Optoelectronic Devices

Eduardo D. Martínez[1*], Carlos D. S. Brites[2], Luís D. Carlos[2], Ricardo R. Urbano[1], Carlos Rettori[1,3]

[1] "Gleb Wataghin" Institute of Physics (IFGW), University of Campinas (UNICAMP), 13083-859, Campinas, SP, Brazil.

[2] Physics Department and CICECO-Aveiro Institute of Materials, University of Aveiro, 3810-193 Aveiro, Portugal.

[3] Federal University of ABC (UFABC), 09210-580, Santo André, SP, Brazil.

*** Correspondence:**

Eduardo D. Martínez, Ph.D.

edmartin@ifi.unicamp.br

1 Structural Characterization 2

2 Temperature Determination using 2S and 2L Nanoparticles 4

Supplementary References 8

1 Structural Characterization

Upconversion nanoparticles (UCNPs) were characterized by x-ray diffraction after synthesis. In all cases, pure hexagonal (β-) phase was obtained consistent with the literature data for pure hexagonal $NaYF_4$ (JCPDS no. 16-0334). Diffractograms with indexed peaks are shown in Supplementary Figure 1. For small-sized UCNPs peaks are broadened due to the small crystallite size and slightly shifted to lower angles due to larger lattice parameters expected for $NaGdF_4$ matrix compared to the $NaYF_4$.

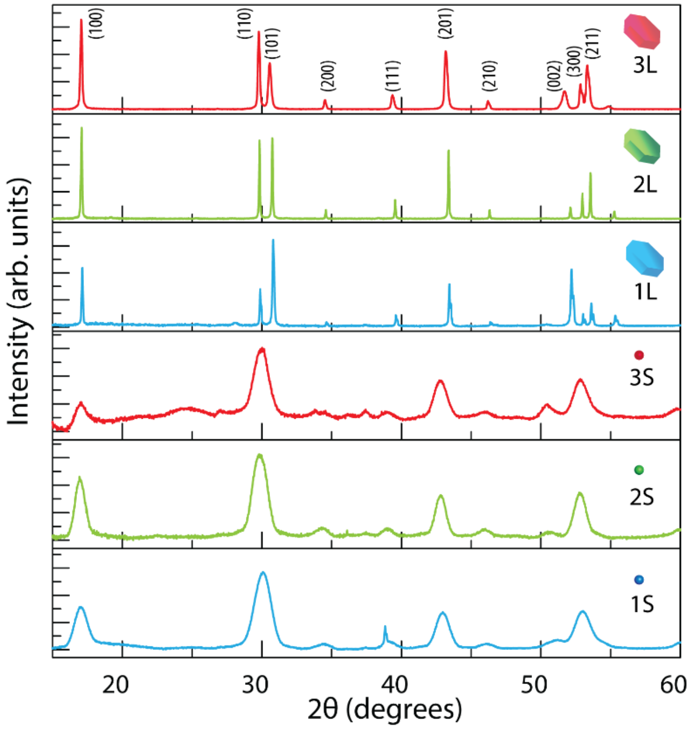

Supplementary Figure 1. XRD of small-sized (RE-$NaGdF_4$) and large-sized (RE-$NaYF_4$) UCNPs doped with different rare-earths (RE).

The particle size distribution of small size UCNPs was determined by image processing of TEM images using ImageJ® 1.48v software. Resulting histograms are displayed in Supplementary Figure 2.

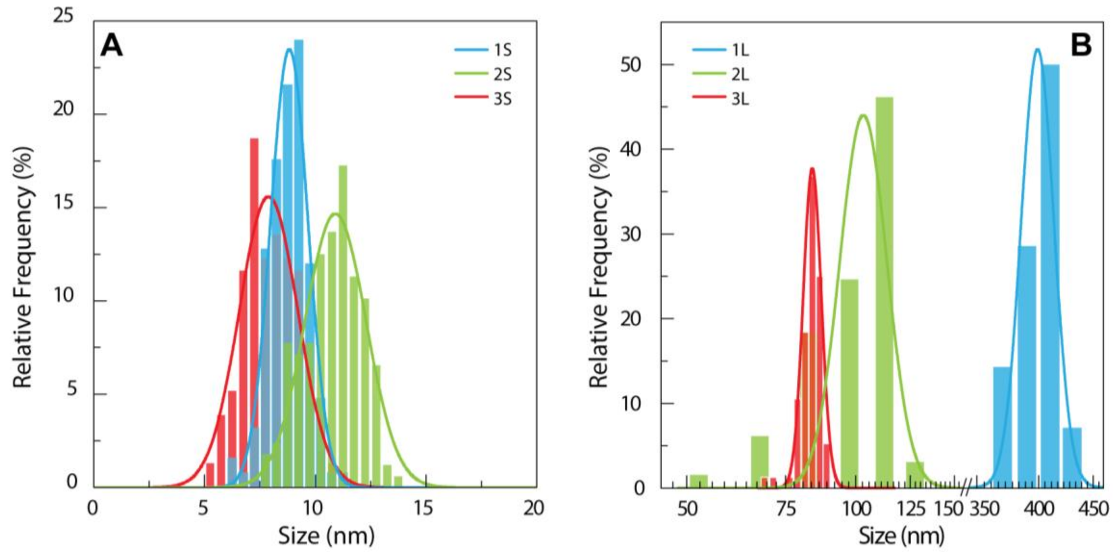

Supplementary Figure 2. Particle size distributions obtained from TEM analysis of (A) small- and (B) large-sized UCNPs.

The characterization of the shell layer in small-size UCNPs was studied for the 2S system as an example. In this study, the particles were characterized before and after the growth of the shell layer by TEM and DLS (Supplementary Figure 3). By calculating the particle size distribution in each case, we obtain an average thickness of (1.6 ± 0.8) nm. As the procedure for the other small-sized UCNPs (1S and 3S) was exactly the same, we estimate similar values for the shell layer in all systems studied.

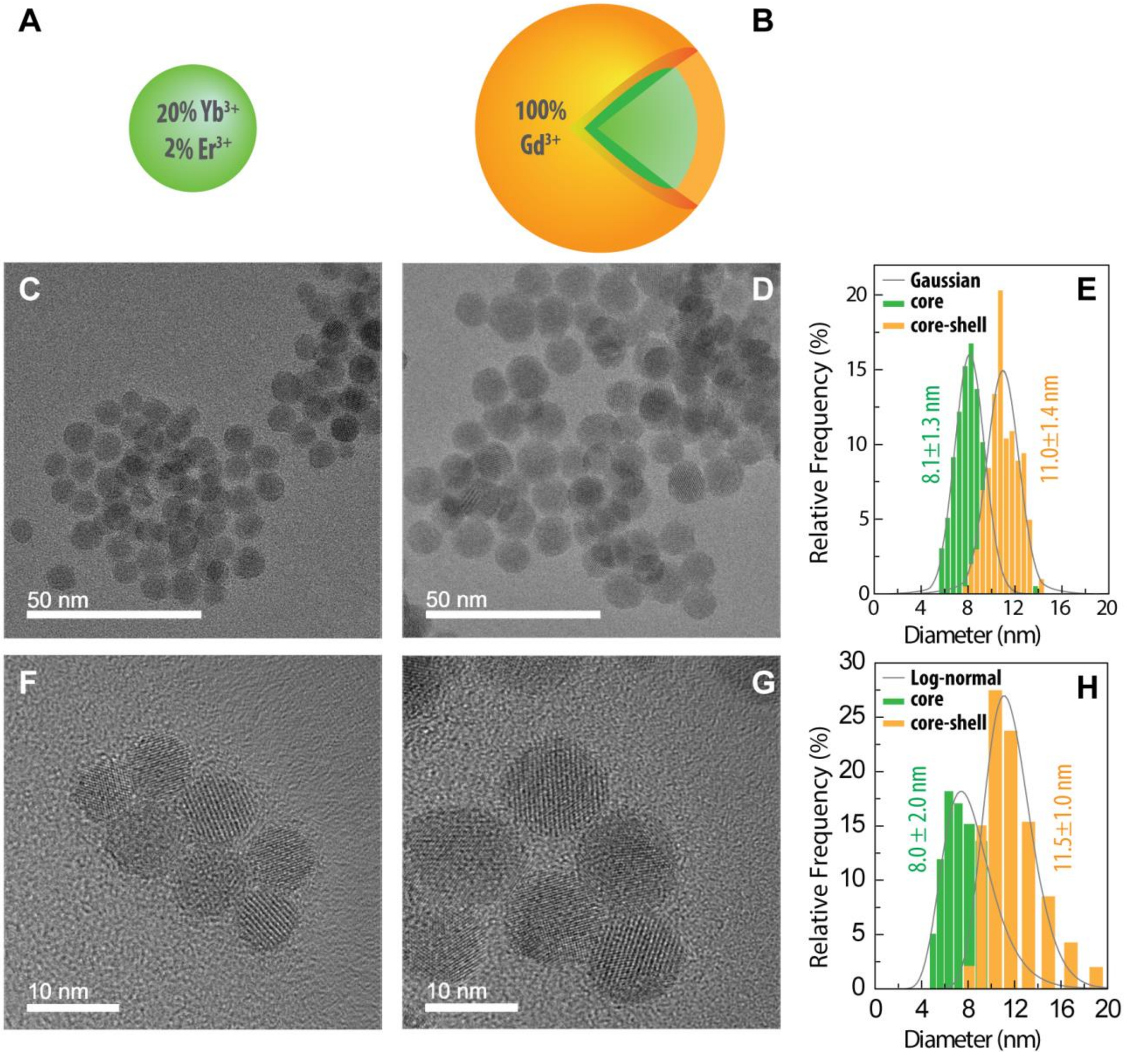

Supplementary Figure 3. Particle size analysis of (A) core and (B) core-shell UCNPs of the set 2S. TEM images of (C, F) core and (D, G) core-shell UCNPs. Particle size distribution calculated by (E) TEM analysis of several images and (F) DLS of UCNPs colloids in cyclohexane.

2 Temperature Determination using 2S and 2L Nanoparticles

Upon 976 nm excitation the overall intensity of the Er^{3+} emission is reduced for the 2L UCNPs and enhanced for 2S UCNPs at increasing temperatures as observed before (Martínez et al., 2018). In both cases, however, the integrated emission intensity arising from the $^2H_{11/2}\rightarrow{}^4I_{15/2}$ transition (I_H) and from the $^4S_{3/2}\rightarrow{}^4I_{15/2}$ transition (I_S) of Er^{3+} can be used as a ratiometric thermometer. For each emission spectrum, the thermometric parameter $\Delta = I_H/I_S$ was calculated according to the procedure illustrated in Supplementary Figure 4A. The local temperature can then be directly calculated from the emission spectra, following the procedures described elsewhere (Brites et al., 2011, 2016; Debasu et al., 2016). The absolute temperature T can be determined by:

$$\frac{1}{T} = \frac{1}{T_0} - \frac{k_B}{\delta E} ln\left(\frac{\Delta}{\Delta_0}\right) \qquad (S1)$$

where k_B is the Boltzmann constant, δE is the energy gap between the $^2H_{11/2}$ and $^4S_{3/2}$ levels, Δ_0 is the thermometric parameter at a known temperature T_0. The Δ_0 value is the thermometric parameter at T_0 in the absence of an excitation laser heating and is estimated as the Δ value at the time instant in which the excitation laser is turned on.

The validity of Equation S1 is clearly shown in Supplementary Figure 4B, in which the thermometric parameters and the corresponding temperatures are represented as an Arrhenius plot for 2S and 2L nanoparticles. By a linear fit, we obtain the slope corresponding to $\delta E/k_B$. We observe an excellent agreement between fitting and data points with correlation values of r^2=0.99992 and 0.9996 for 2S and 2L, respectively. The resulting values are listed in Supplementary Table 1.

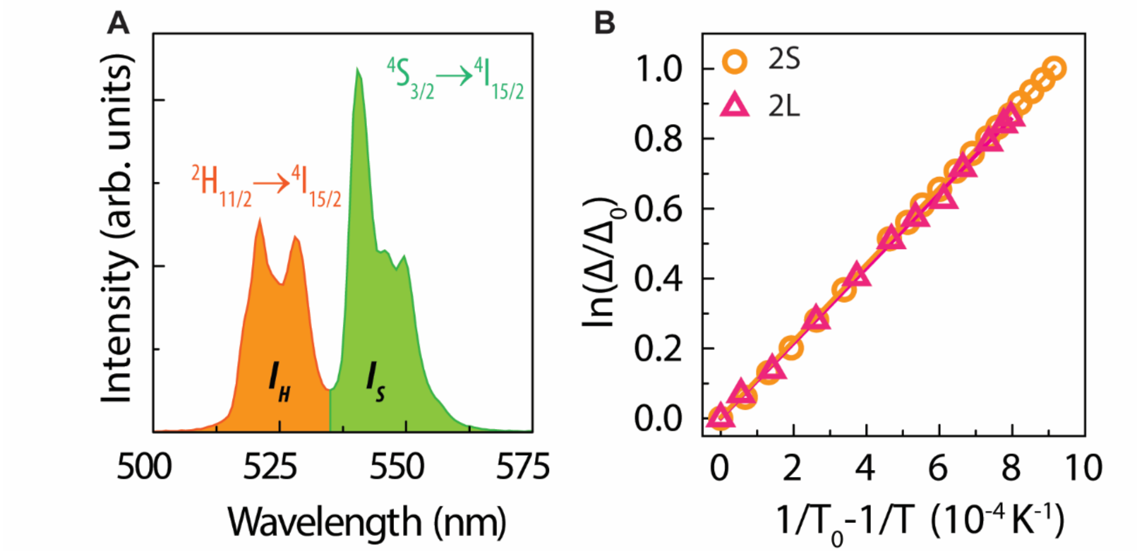

Supplementary Figure 4. Thermometric characterization of Er^{3+}-doped UCNPs. (A) Integrated bands for the calculation of the thermometric parameter Δ, (B) linearized relation and fitting for the determination of δE.

Supplementary Table 1. Parameters used for temperature determination using Equation S1 for samples 2S and 2L.

Parameter	2S	2L
Δ_0	0.29 ± 0.01	0.26 ± 0.01
T_0 (K)	296 ± 1	297 ± 1
δE (cm^{-1})	760 ± 2	744 ± 4
r^2	0.99992	0.9996

3 Thermal properties of additional UCNPs

The thermal dependence of the emission spectra for additional UCNPs studied in this work is presented in Supplementary Figure 5. Measurements were performed by drop-casting the UCNPs colloids in hexane or cyclohexane on silicon wafers. After evaporation of the solvent at 130 °C, samples were placed on a Peltier plate controlled by an Arduino board. The emission spectra were acquired at varying temperatures of the plate under 976 nm laser excitation. Power densities ranged from 10 to 50 W·cm^{-2} were used, while the acquisition time varied from 5 to 15 s, depending on the amount of UCNPs deposited and their luminescence efficiency.

As observed in Supplementary Figure 5, the emission intensity is thermally quenched for large-sized UCNPs doped either Yb^{3+}-Tm^{3+} (1L) or Yb^{3+}-Ce^{3+}-Ho^{3+} ions (3L). On the contrary, thermal enhanced emission is observed for small-sized UCNPs with the same composition (1S and 3S). The thermal effects on the emission intensity were summarized by defining the relative integrated intensity (RII) as the integrated spectra at a given temperature divided by the integrated spectra obtained at room (initial) temperature. For UCNPs doped with Yb^{3+}-Er^{3+} (2L and 2S) the emission spectra was shown in Figures 1D,H of the main text, respectively. In Supplementary Figures 5E,F, we present the RII calculated for those samples.

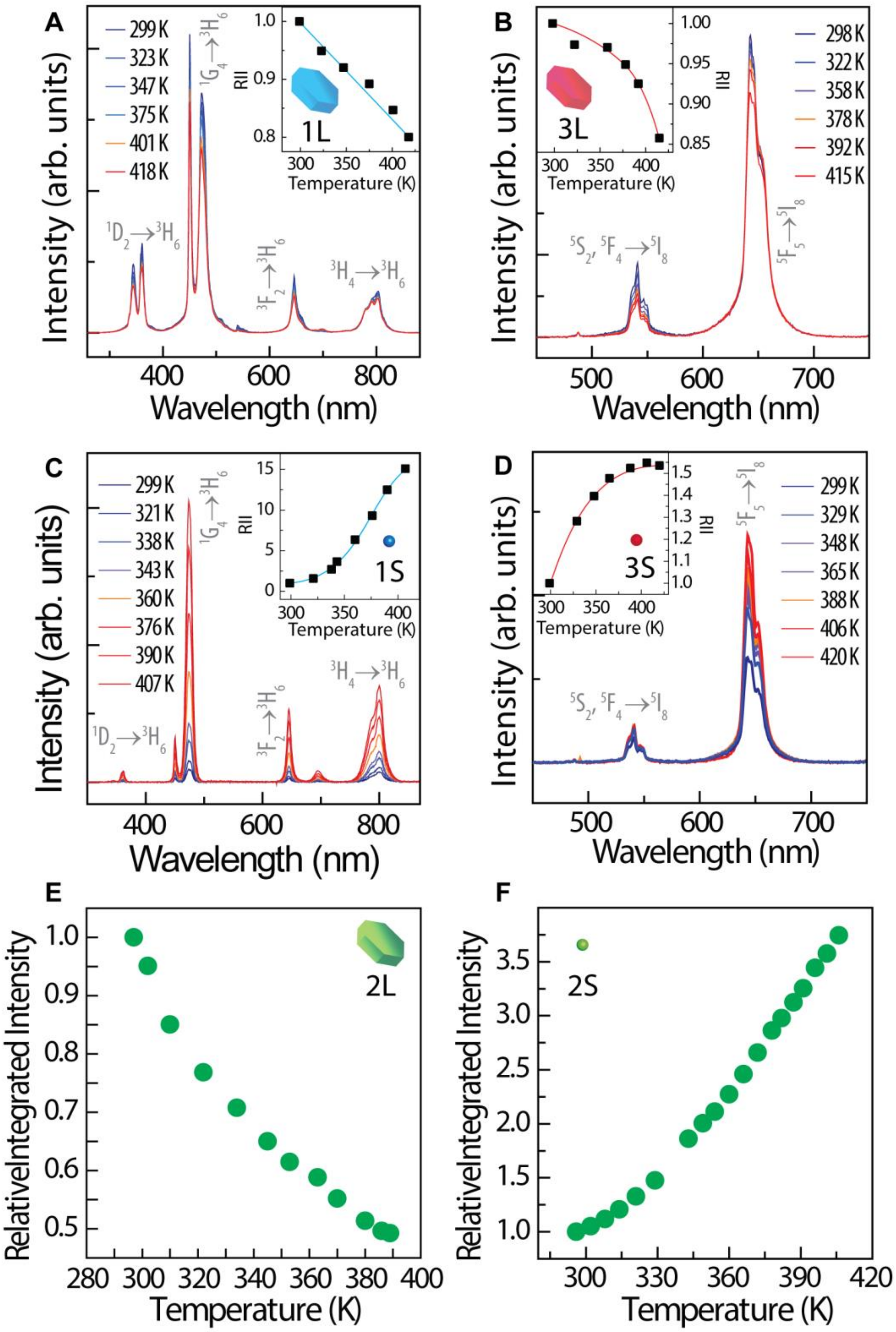

Supplementary Figure 5. Temperature dependent emission spectra of (A) 1L, (B) 3L, (C) 1S and (D) 3S upon 976 nm excitation. Relative integrated intensity (RII) are shown as insets. Data of RII corresponding to UCNPs of the set 2L and 2S are shown in panels (E) and (F), respectively.

Supplementary References

Brites, C. D. S., Lima, P. P., Silva, N. J. O., Millán, A., Amaral, V. S., Palacio, F., et al. (2011). Lanthanide-based luminescent molecular thermometers. *New J. Chem.* 35, 1177. doi:10.1039/c0nj01010c.

Brites, C. D. S., Millán, A., and Carlos, L. D. (2016). Lanthanides in Luminescent Thermometry. *Handb. Phys. Chem. Rare Earths* 49, 339–427. doi:10.1016/bs.hpcre.2016.03.005.

Debasu, M. L., Brites, C. D. S., Balabhadra, S., Oliveira, H., Rocha, J., and Carlos, L. D. (2016). Nanoplatforms for Plasmon-Induced Heating and Thermometry. *ChemNanoMat* 2, 520–527. doi:10.1002/cnma.201600061.

Martínez, E. D., Urbano, R. R., and Rettori, C. (2018). Thermoplasmonic enhancement of upconversion in small-size doped $NaGdYF_4$ nanoparticles coupled to gold nanostars. *Nanoscale* 10, 14687–14696. doi:10.1039/c8nr01639a.
